# Radiobiology of Proton Therapy in Human Papillomavirus-Negative and Human Papillomavirus-Positive Head and Neck Cancer Cells

**DOI:** 10.3390/cancers16111959

**Published:** 2024-05-22

**Authors:** Rüveyda Dok, Laura Vanderwaeren, Kevin J. Verstrepen, Sandra Nuyts

**Affiliations:** 1 Laboratory of Experimental Radiotherapy, Department of Oncology, KU Leuven, University of Leuven, 3000 Leuven, Belgium; 2 Laboratory for Systems Biology, VIB-KU Leuven Center for Microbiology, 3000 Leuven, Belgium; 3 Laboratory of Genetics and Genomics, Centre for Microbial and Plant Genetics, KU Leuven, 3000 Leuven, Belgium; 4 Department of Radiation Oncology, Leuven Cancer Institute, UZ Leuven, 3000 Leuven, Belgium

**Keywords:** head and neck cancer, photon radiotherapy, proton therapy, radiobiology

## Abstract

**Simple Summary:**

Our understanding of the underlying biology of proton therapy in head and neck cancers is limited. Here, we assessed the proton therapy-related radiobiological processes in head and neck cancer cells. We showed that proton therapy resulted in biological effects similar to those of classical photon-based radiotherapy.

**Abstract:**

Photon-based radiotherapy (XRT) is one of the most frequently used treatment modalities for HPV-negative and HPV-positive locally advanced head and neck squamous cell carcinoma (HNSCC). However, locoregional recurrences and normal RT-associated toxicity remain major problems for these patients. Proton therapy (PT), with its dosimetric advantages, can present a solution to the normal toxicity problem. However, issues concerning physical delivery and the lack of insights into the underlying biology of PT hamper the full exploitation of PT. Here, we assessed the radiobiological processes involved in PT in HPV-negative and HPV-positive HNSCC cells. We show that PT and XRT activate the DNA damage-repair and stress response in both HPV-negative and HPV-positive cells to a similar extent. The activation of these major radiobiological mechanisms resulted in equal levels of clonogenic survival and mitotic cell death. Altogether, PT resulted in similar biological effectiveness when compared to XRT. These results emphasize the importance of dosimetric parameters when exploiting the potential of increased clinical effectiveness and reduced normal tissue toxicity in PT treatment.

## 1. Introduction

Head and neck squamous cell cancer (HNSCC) is the sixth most frequent cancer worldwide. It is currently divided into two groups according to their etiology, namely, alcohol- and tobacco-related cancers and human papillomavirus (HPV)-related cancers. Although HPV-positive HNSCC is characterized by a better treatment outcome compared to HPV-negative HNSCC, treatment of HNSCC is not yet determined by HPV status [1,2,3,4]. Especially, recent efforts in de-escalation trials, including the use of transoral robotic surgery (TORS) followed by risk-adjusted adjuvant therapy, highlight the importance of HPV determination [5,6].

Radiotherapy (RT) in combination with chemotherapy is one of the most frequently used treatment modalities for both HPV-negative and HPV-positive locally advanced HNSCC. The delivery and planning of conventional or photon-based RT (XRT) has shown tremendous technological advances in the past few decades, resulting in targeted deliveries of radiation and improved treatment outcomes [1,7,8,9].

Nonetheless, recurrences and treatment-induced normal tissue toxicity remain major problems for patients with HNSCC. The vast majority of recurrences are locoregional, highlighting the need for improved treatment strategies in order to improve the treatment outcome while also limiting normal tissue toxicity [1,2,3,8,9].

The normal tissue toxicity of XRT can be related to the relatively high entrance and exit doses of photons. Particles are slowed down and deposit most of their energy at the end of their range, resulting in a very characteristic depth–dose distribution called the Bragg peak, potentially reducing the radiation exposure to surrounding normal tissues, compared to XRT [10,11]. This physical property, and the associated potential clinical benefit, have led to the opening of several PT centers worldwide [12]. Although PT can be used to treat numerous cancer patients, including HNSCC patients, there are still major hurdles to overcome in terms of physical delivery and insights in the radiobiology of PT [11].

In order to account for and to compare the effectiveness of PT to XRT, the relative biological effectiveness (RBE) is used. RBE is the ratio of doses of a reference relative to a test radiation producing the same biological radiation effect. Currently, in clinical practice, as recommended by the International Commission on Radiation Units and Measurements (ICRU), a constant RBE value of 1.1 is used for PT in all tissues and across the entire irradiated volume. Numerous studies showed, however, variable RBE values in different biological systems [13,14]. The use of a consensus RBE is often justified, given the uncertainties in the available RBE data.

The radiation-induced biological response depends on various physical parameters including the radiation type, the loss of energy per unit-path length, also called linear energy transfer (LET), the dose, the dose rate, the dose fraction, biological parameters such as the cell and tissue type, the radiobiological processes and the biological endpoint that is measured. The effects of physical properties on RBE are well understood, whereas the biological properties are understudied. Hence, the debate about whether to change the current clinical practice of using a fixed RBE of 1.1 is still ongoing [15,16,17].

Here, we investigate the radiobiological effects of proton therapy in different subgroups of HNSCC to shed light on the molecular pathways that determine the RT response.

## 2. Materials and Methods

### 2.1. Cell Culture

Four HNSCC cell lines were used. These were HPV-positive UPCI-SCC-154 (DSMZ, Braunschweig, Germany), HPV-positive UM-SCC-104 (given by Dr T. Carey, University of Michigan, Ann Arbor, MI, USA), HPV-negative SQD9 and HPV-negative CAL27 (both given by Dr. A. Begg from the Netherlands Cancer Institute, Amsterdam, The Netherlands) cell lines.

Minimal Essential Medium (MEM, Thermo Fisher Scientific, Waltham, MA, USA), supplemented with 10% fetal bovine serum (FBS, Thermo Fisher Scientific), 1% L-glutamine (Thermo Fisher Scientific) and 1% non-essential amino acids (Thermo Fisher Scientific) was used for HPV-positive cells. Dulbecco’s Modified Eagle Medium (DMEM, Thermo Fisher Scientific), supplemented with 10% FBS and 1% sodium pyruvate (Thermo Fisher Scientific) was used for HPV-negative cells.

All cells were mycoplasma-free and short-tandem repeat profiling (ATCC) was performed for the cell lines used. The experiments were performed with approval from the Ethics Committee Research UZ/KU Leuven (S63252; approval date 18 December 2019).

XRT was delivered with SARRP (220 kV photons, 13 mA, X-Strahl, Camberley, UK, dose rate: 4 Gy Gy/min). Proton irradiations (62 MeV, mean LET = 10.51 keV/µm) were performed at the Centre-de-Ressources du Cyclotron (Université Catholique de Louvain, Louvain-la-Neuve, Belgium). Samples were placed at the entrance of the proton depth–dose curve for irradiations.

### 2.2. Colony Assay

Cells were irradiated with RT doses varying from 0–6 Gy. After 2–3 weeks, cells were fixed with 2.5% glutaraldehyde in PBS and stained with 0.4% crystal violet. The colonies containing 50 cells or more were counted with GelCount colony counter (Oxford Optronix, Abington, UK). The survival fractions were calculated by normalizing to the plating efficiency of the non-irradiated controls specific to that treatment modality. Plating efficiencies were determined by dividing the number of colonies counted by the number of cells initially seeded.

### 2.3. Flow Cytometry

Cells were fixed with 70% ethanol and 10 µg/mL propidium iodide (P4170, Sigma-Aldrich, Saint Louis, MO, USA) containing 100 µg/mL RNAse A (12091021, Invitrogen, Carlsbad, CA, USA) was used for staining. Cell cycle was assessed by FACSverse (BD Biosciences, San Jose, CA, USA).

### 2.4. Immunofluorescence

Cells were seeded in µClear 96-well plates (Greiner Bio-one, Kremsmünster, Austria). For fixation, 4% paraformaldehyde was used, and for permeabilization, 0.3% Triton X-100 (Sigma-Aldrich, Saint-Louis, MO, USA) in 0.5% BSA-PBS was used. Cells were stained with a primary antibody against pH2AX Ser139 (JBW301, Millipore, Burlington, MA, USA), and then with an Alexa Fluor 488 secondary antibody (4408 CST, Danvers, MA, USA). DAPI (D9542, Sigma-Aldrich, Saint-Louis, MO, USA) was used for nuclei staining. Immunofluorescence images were acquired with an Operetta CLS High Content Analysis System (PerkinElmer, Waltham, MA, USA) and analyzed with image J version 1.54 (Rasband W. and contributors, National Institute of Health, USA).

### 2.5. Western Blotting

Cells were irradiated with 6 Gy and were lysed using RIPA buffer. The lysis buffer contained sodium vanadate phosphatase (S6508, Sigma-Aldrich, Saint Louis, MO, USA) and cOmplete™, an EDTA-free Protease Inhibitor Cocktail (11873580001, Sigma-Aldrich, Saint Louis, MO, USA). SeeBlue™ (LC5925, Invitrogen, Carlsbad, CA, USA) was used as the protein standard. Blocking was performed with 5% non-fatty dry milk. The primary antibodies for p-PER (#3179, CST, Danvers, MA, USA), ATF (#11815, CST, Danvers, MA, USA), eIF2α (#9722, CST, Danvers, MA, USA), p-eIF2α (#9721, CST, Danvers, MA, USA), p16 (clone G175-405, BD Pharmingen, Franklin Lakes, NJ, USA) and vinculin (clone hVIN-1, V9131, Sigma-Aldrich, Saint Louis, MO, USA) were incubated overnight at 4 °C. For secondary antibodies, anti-mouse IgG (#7076, CST, Danvers, MA, USA) and anti-rabbit IgG (#7074, CST, Danvers, MA, USA) were used.

### 2.6. Statistical Analysis

Statistical analysis was performed with GraphPad software version 6 (San Diego, CA, USA), with all tests deemed statistically significant at *p* < 0.05.

## 3. Results

### 3.1. Effect of PT and XRT on Survival of HNSCC Cells

**Figure 1:**
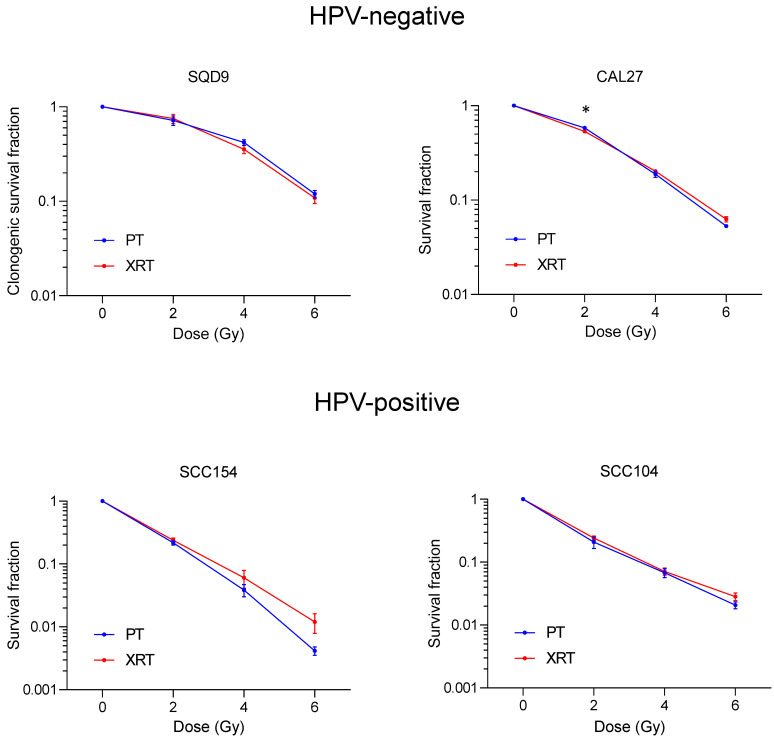
Effects of PT and XRT on clonogenic survival of HNSCC cells. Clonogenic survival of HPV-negative (SQD9 and CAL27) and HPV-positive (SCC154 and SCC104) cells after treatment with PT or XRT. Data is shown as the mean ± s.e.m., n = 3. *p*-values were calculated with two-way ANOVA with correction for multiple testing. PT = proton therapy, XRT = X-rays. * *p*-values < 0.05 were calculated with ANOVA with multiple comparisons test.

To investigate the effect of PT on survival in HPV-positive and HPV-negative HNSCC cells, we performed a clonogenic survival assay. In line with previous studies [18,19,20], HPV-positive cells showed a higher radiosensitivity compared to HPV-negative cells. Treatment with 2 Gy PT resulted in an average survival fraction of 0.72 (s.e.m ± 0.08) for HPV-negative SQD9 cells and an average survival fraction of 0.58 (s.e.m ± 0.01) for HPV-negative CAL27 cells (Figure 1). In HPV-positive cells, 2 Gy PT resulted in average survival fractions of 0.22 (s.e.m ± 0.02) and 0.21 (s.e.m ± 0.04) for SCC154 and SCC104, respectively (Figure 1. When compared to XRT, the clonogenic survival of HPV-negative SQD9 and HPV-positive SCC104 cells did not significantly differ in PT. In HPV-negative CAL27 cells, 2 Gy XRT resulted in a slight but significant decrease in clonogenic survival (0.53 s.e.m ± 0.02), compared to PT. In contrast, HPV-positive SCC154 cells showed a higher efficacy in cell kill at 4 and 6 Gy, with average survival fractions of 0.04 (s.e.m ± 0.008) and 0.004 (s.e.m ± 0.001) for PT and average survival fractions of 0.06 (s.e.m ± 0.02) and 0.01 (s.e.m ± 0.004) for XRT, respectively. However, these differences were not statistically significant (Figure 1).

**Figure 2:**
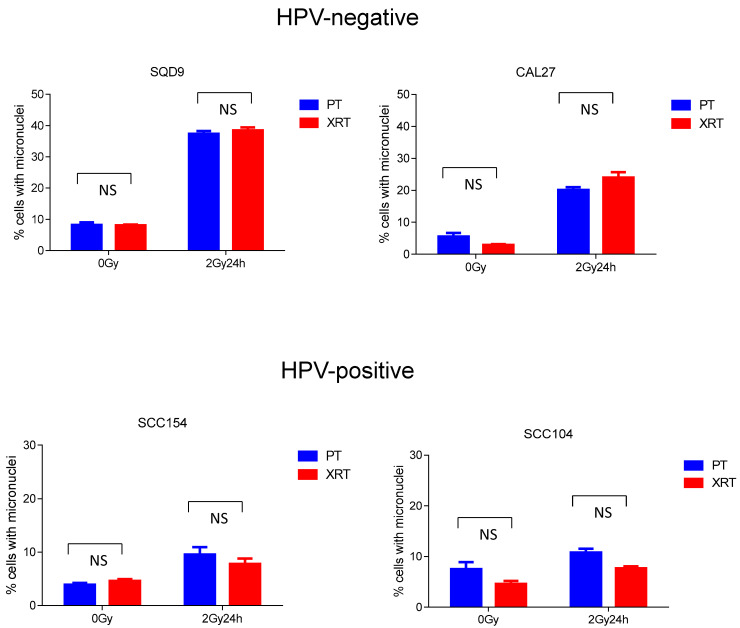
Effects of PT and XRT on micronuclei formation. HPV-negative (SQD9 and CAL27) and HPV-positive (SCC154 and SCC104) cells were exposed to 2 Gy PT or XRT and the percentage of micronucleated cells to the total number of cells was determined. Data is shown as the mean ± s.e.m., n = 3. *p*-values were calculated with two-way ANOVA with correction for multiple testing. PT = proton therapy, XRT = X-rays, NS = non-significant.

Previously it was shown that PT can induce cell death at a higher extent compared to XRT [21]. Since the majority of HNSCC cells undergo radiation-induced mitotic cell death [21,22], we assessed the importance of mitotic cell death in PT by determining the number of micronucleated cells (Figure 2). PT treatment with 2 Gy resulted in average values of 37% (s.e.m ± 0.88) and 20% (s.e.m ± 0.90) micronucleated cells in HPV-negative SQD9 and CAL27, respectively. In contrast, in SCC154 and SCC104 cells, 2 Gy PT resulted in average values of 10% (s.e.m ± 1.40) and 11% (s.e.m ± 0.73) for micronucleated cells, respectively. No significant differences in the percentages of micronucleated cells were detected between PT and XRT as to all HNSCC cells. In HPV-negative SQD9 and CAL27 cells 2 Gy XRT resulted in average values of 38% (s.e.m ± 0.97) and 24% (s.e.m ± 1.70) of micronucleated cells. For HPV-positive SCC154 and SCC104, an average of 8% (s.e.m ± 1.01 for SCC154 and s.e.m ± 0.43 for SCC104) of micronucleated cells were detected.

At 0 Gy, HPV-negative CAL27 and HPV-positive SCC104 cells showed a non-significant difference in the percentage of micronucleated cells between PT (6% s.e.m ± 1.09 for CAL27 and 8% s.e.m ± 1.37 for SCC104) and XRT (3% s.e.m ± 0.26 for CAL27 and 5% s.e.m ± 0.63 for SCC104) conditions.

### 3.2. Effect of PT and XRT on DNA Damage Repair and Cell Cycle Kinetics

**Figure 3:**
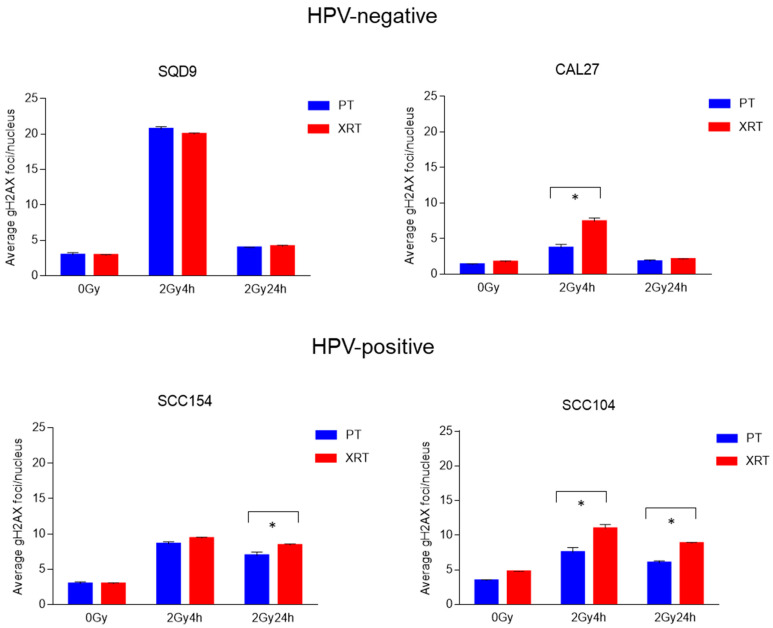
Effects of PT and XRT on DNA double strand repair kinetics. Average number of γH2AX foci per cell was determined in control 0 Gy and 2 Gy treated cells at indicated time points after an RT dose of 2 Gy. Data are presented as the mean ± SEM for n = 3. * *p*-values < 0.05 were calculated with ANOVA with multiple comparisons test. PT = proton therapy, XRT = X-rays.

**Figure 4:**
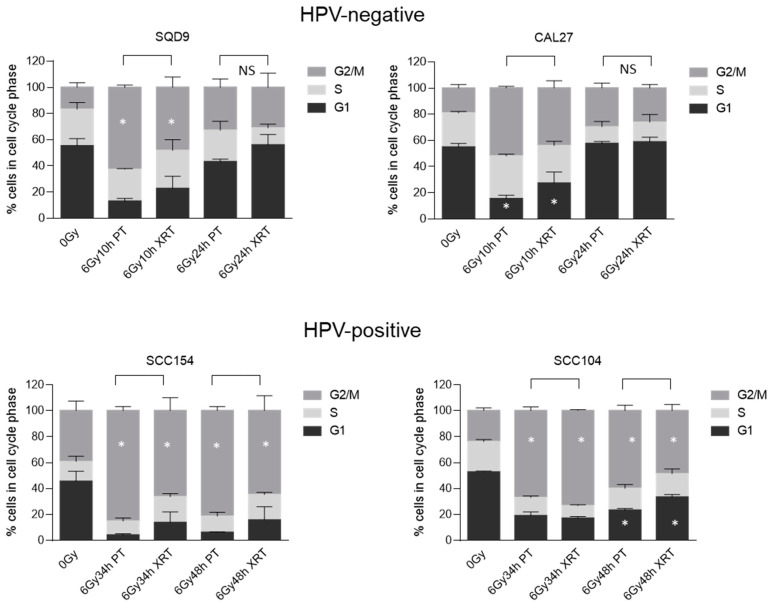
Effects of PT and XRT on cell cycle progression. Cell cycle distribution of HPV-negative SQD9 and CAL27 and HPV-positive SCC154 and SCC104 cells in G1, S and G2/M phases in 0 Gy (control) and 6 Gy conditions, at different time points. Data are presented as the mean ± SEM for n = at least 3. * *p*-values < 0.05 were calculated with ANOVA with multiple comparisons test. PT = proton therapy, XRT = X-rays, NS = non-significant.

Radiation-induced DNA damage repair is crucial for the survival of cells, irrespective of the radiation source. However, differences in the extent and duration of radiation-induced DNA damage have been reported after treatment with PT [23,24,25,26,27,28,29]. Therefore, DNA damage repair kinetics were determined by assessing the number of γH2AX foci (Figure 3) and cycle progression (Figure 4).

Corresponding with the intrinsic radiosensitivity, HPV-positive cells showed a higher number of residual γH2AX compared to HPV-negative cells (Figure 3). In addition, small but significant differences in the number of residual γH2AX foci can be seen between PT and XRT treatment in HPV-positive cells. For HPV-positive SCC154, average values of 7 (s.e.m ± 0.52) and 8 (s.e.m ± 0.20) γH2AX foci were detected at 24 h after 2 Gy of PT and XRT, respectively. HPV-positive SCC104 cells showed at 24 h after PT and XRT average values of 6 (s.e.m ± 0.32) and 9 (s.e.m ± 0.12) γH2AX foci, respectively. In HPV-positive SCC104, significant differences at 4 h after irradiation were detected, with average values of 8 (s.e.m ± 0.68) and 11 (s.e.m ± 0.59) γH2AX foci for PT and XRT, respectively.

In contrast, HPV-negative SQD9 and CAL27 cells showed no residual γH2AX foci after 2 Gy 24 h PT or XRT. SQD9 cells showed an average of 4 residual γH2AX foci for PT (s.e.m ± 0.07) and XRT (s.e.m ± 0.14). CAL27 cells showed an average of 2 residual γH2AX foci for both PT (s.e.m ± 0.25) and XRT (s.e.m ± 0.09). CAL27 cells exhibited a significantly higher number of γH2AX foci at 4 h after XRT (7 s.e.m ± 0.49) compared to PT (4 s.e.m ± 0.54).

In line with DNA damage-repair kinetics, HPV-positive cells show a prolonged G2/M arrest (Figure 4). In contrast, the G2/M arrest of HPV-negative cells was less pronounced and showed a faster clearance. Small but significant differences were detected in cell cycle progression. HPV-negative SQD9 cells show a significant difference between the two radiation sources, with 63% (s.e.m ± 1.00) and 48% (s.e.m ± 4.60) cells showing a G2/M arrest in PT- and XRT-treated conditions, respectively. Contrastingly, CAL27 cells showed a significant difference in the number of cells accumulated in the G1 phase, with average accumulations of 15% (s.e.m ± 1.55) and 27% (s.e.m ± 4.96) in PT- and XRT-treated conditions, respectively. Despite this, no substantial differences in cell cycle kinetics were seen in HPV-negative cells between PT and XRT treatment conditions.

HPV-positive SCC154 cells show at 34 h and 48 h after PT treatment a more pronounced G2/M arrest, as compared to XRT treatment (Figure 4). For SCC154 cells the difference between PT and XRT was on average 19% and 16% for 34 h and 48 h, respectively. For HPV-positive SCC104 cells, significant differences were seen in the G2/M phase at 34 and 48 h after RT. More specifically, on average, 67% and 60% of cells were detected in G2/M phase in 34 h and 48 h after PT, whereas these values were 73% and 49% in XRT-treated conditions.

### 3.3. Effect of PT and XRT on Stress Response

**Figure 5:**
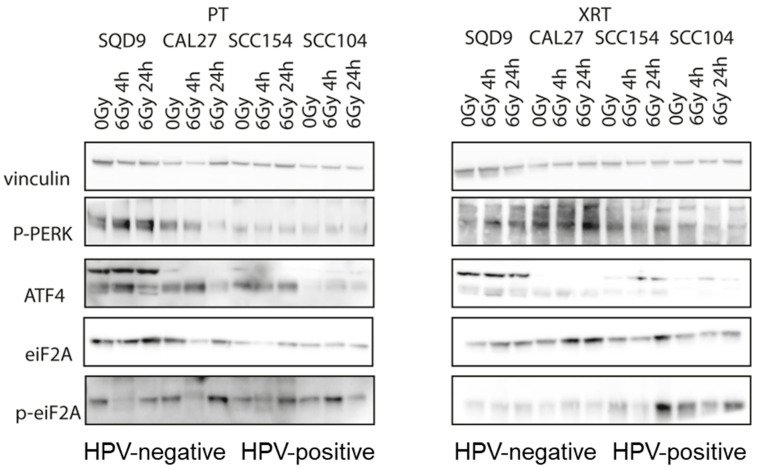
Effects of PT and XRT on stress response. Protein levels of p-PERK (170 kDa), ATF4 (49 kDa), eIF2α (38 kDa), p-eIF2α (38 kD) and Vinculin (124 kDa) in HPV-negative SQD9 and CAL27 and HPV-positive SCC154 and SCC104 cells in control 0 Gy and in irradiated 6 Gy PT, or RT conditions. At the indicated time points, total cell lysates were prepared. kDa = molecular weight as determined by the protein standard. PT = proton therapy, XRT = X-rays.

Aside from DNA damage, radiation is accompanied with a stress response on the transcriptomic and proteomic levels [30]. To determine whether PT alters the stress response in HNSCC cells, we assessed the expression of several key stress response proteins. Phospho-protein kinase-like endoplasmic reticulum kinase (p-PERK) is known to be activated during endoplasmic reticulum (ER) stress and phosphorylates eukaryotic initiation factor 2 α (eIF2 α) subunit to reduce protein translation. Activating Transcription Factor 4 (ATF-4) is known to increase the expression of genes critical for the recovery from ER stress. Although P-PERK, p-eIF2α are activated at various levels after PT or XRT, the changes are cell line-specific and not related to treatment modality (Figure 5, Figures S1 and S2).

## 4. Discussion

PT is emerging as an alternative radiation treatment modality with the potential to reduce normal tissue toxicity issues seen in XRT treatments. Several studies comparing intensity-modulated proton therapy (IMPT) to intensity-modulated radiation therapy (IMRT) in HNSCC have demonstrated both dosimetric and potential clinical benefits of IMPT [31,32,33,34,35,36,37]. Consistent with this, a recent retrospective study involving patients with oropharyngeal carcinoma treated with intensity-modulated proton therapy (IMPT) demonstrated significantly lower levels of acute toxicity compared to those treated with intensity-modulated radiation therapy (IMRT). Regarding the chronic adverse effects, only xerostomia showed a significant difference between IMPT-treated patients and those treated with IMRT [37]. Furthermore, numerous randomized trials are currently underway to validate the efficacy of PT in reducing toxicity to normal tissues [38].

The benefit of PT is particularly associated with the dosimetric properties of particle-based radiation. Several studies have investigated the radiobiological response of PT and compared it to XRT using an in vitro HNSCC model. These studies show an enhanced biological effectiveness in PT ranging from 1.06–1.54, highlighting the presence of biological variation and the importance of radiation parameters such as dose rates, energies and LET [11,15,21,24,25,27,28,29,30,38,39,40,41,42,43,44,45,46]. Understanding radiobiological processes will clarify the biological uncertainties accompanying PT, but it will also stimulate the development of PT-specific combinatorial strategies.

We recently performed an in-depth comparison of PT- and XRT-induced radiobiological processes in S. cerevisiae [30]. One of the striking differences between PT and XRT treatment in S. cerevisiae was on the transcriptomic level, with PT showing a stronger activation of genes involved in proteotoxic stress response. Although we could detect the activation of several key stress response proteins in both HPV-positive and HPV-negative HNSCC cells, the activation was not dependent on the radiation source. These data indicate that although S. cerevisiae is a good model to study radiobiological processes, differences in dose and biological endpoint should be considered when translating findings from model organisms to human cancer cells. To date the knowledge about PT-related protein and gene expression changes in HNSCC are limited. However, differences in genes involved in angiogenesis, cell proliferation and metastasis have been reported between PT and XRT [24,26,47].

Another process that is not only a detrimental factor in XRT response but also differentially regulated between the two groups of HNSCC is DNA damage repair. It is well known that HPV-positive HNSCC cells are characterized by decreased DNA damage repair capacity [19,44,48,49,50,51]. In agreement with this, HPV-positive HNSCC cells treated with PT showed prolonged activation of G2/M arrest, which was accompanied with residual γH2AX foci when compared to HPV-negative cells. This difference was also reflected in clonogenic survival differences between HPV-positive and HPV-negative HNSCC cells.

Several studies have shown that PT can induce more complex DNA lesions, resulting in differential activation of DNA repair processes and mitotic cell death compared to XRT [13,14,16,17,21,28,41]. In contrast to this, we showed that in HNSCC cells the DNA damage repair and cell cycle kinetics for PT and XRT were similar. It should be noted that cell line-dependent differences in DNA damage repair and cell cycle kinetics were detected between the two radiation sources. However, these did not lead to significant differences in survival. Despite lacking significance, treatment with 4 and 6 Gy of PT resulted in lower clonogenic survival in HPV-positive SCC154 cells compared to XRT. Wang et al. previously reported that HPV-positive cells were more sensitive to PT than XRT [25]. This difference in biological effectiveness was cell type-dependent, which might explain our results. Nonetheless, Wang et al. also indicate that the increased effectiveness of PT was dependent on radiation fraction size, with lower fractions demonstrating a higher level of radiobiological effects [25]. This contrasts with our findings, as we only observed differences at higher radiation fraction sizes.

In line with the clonogenic survival data, we did not observe any significant differences in micronucleated cells after PT or XRT. Lower numbers of micronucleated cells were seen in HPV-positive cells compared to HPV-negative cells. Since HPV-positive cells show prolonged G2/M arrest up to 48 h after irradiation, the lower number of micronucleated number cells can be explained by the timepoint, namely 24 h after irradiation, in which the micronuclei were assessed.

The discrepancy between this study and studies showing stronger biological effectiveness might be explained by the dose–depth profile of our PT treatment experiments. To ensure uniform dose distribution, our PT experiments were performed in the plateau region of the depth–dose profile, resulting in lower LET compared to irradiation in the Bragg peak or spread-out Bragg peak (SOBP). As discussed before, irradiation in SOBP or in the Bragg peak can increase the extent of DNA damage [13,14].

Despite this limitation, we can conclude that our data indicate that both PT and RT show similar radiobiological responses in 2 HPV-negative and 2 HPV-positive HNSCC cell lines. As previously noted, investigations into the radiobiology of PT have demonstrated considerable variability in RBE. We believe that our study will contribute to the existing body of literature and will help determine the magnitude of this biological diversity and its potential implications in clinical contexts. Moreover, we believe that our study is particularly noteworthy, as it suggests that physical parameters will be the major determinants of clinical effectiveness, and this should be taken into account in PT treatments. In line with this, our results suggest that only a lower integral radiation dose may lead to reduced toxicities compared to RT treatment strategies. However, we acknowledge that further validation of our results is necessary through experiments assessing the biological effectiveness of PT using different LET.

## 5. Conclusions

Altogether, our study shows that PT and XRT result in comparable biological effectiveness, highlighting the importance of dosimetric parameters in exploiting the potential for increased clinical effectiveness and decreased normal tissue toxicity with PT treatment.

## Data Availability

The data presented in this study can be made available upon request from the corresponding author.

## Supplementary Materials

The following supporting information can be downloaded at: https://www.mdpi.com/article/10.3390/cancers16111959/s1, Figure S1: Uncropped western blot images of HNSCC cells treated with PT used in Figure 5. Proteins used in Figure 5.are indicated with red boxes. Protein ladder: SeeBlue™ (LC5925, Invitrogen, Carlsbad, CA, USA).; Figure S2: Uncropped western blot images of HNSCC cells treated with XRT used in Figure 5. Proteins used in Figure 5 are indicated with red boxes. Protein ladder: SeeBlue™ (LC5925, Invitrogen, Carlsbad, CA, USA).

## References

[1] Algazi A.P., Grandis J.R. (2017). Head and neck cancer in 2016: A watershed year for improvements in treatment?. Nat. Rev. Clin. Oncol..

[2] Alsahafi E., Begg K., Amelio I., Raulf N., Lucarelli P., Sauter T., Tavassoli M. (2019). Clinical update on head and neck cancer: Molecular biology and ongoing challenges. Cell Death Dis..

[3] Cramer J.D., Burtness B., Le Q.T., Ferris R.L. (2019). The changing therapeutic landscape of head and neck cancer. Nat. Rev. Clin. Oncol..

[4] Johnson D.E., Burtness B., Leemans C.R., Lui V.W.Y., Bauman J.E., Grandis J.R. (2020). Head and neck squamous cell carcinoma. Nat. Rev. Dis. Prim..

[5] Ferris R.L., Flamand Y., Weinstein G.S., Li S., Quon H., Mehra R., Garcia J.J., Chung C.H., Gillison M.L., Duvvuri U. (2022). Phase II Randomized Trial of Transoral Surgery and Low-Dose Intensity Modulated Radiation Therapy in Resectable p16+ Locally Advanced Oropharynx Cancer: An ECOG-ACRIN Cancer Research Group Trial (E3311). J. Clin. Oncol..

[6] Mehanna H., Rischin D., Wong S.J., Gregoire V., Ferris R., Waldron J., Le Q.-T., Forster M., Gillison M., Laskar S. (2020). De-Escalation After DE-ESCALATE and RTOG 1016: A Head and Neck Cancer InterGroup Framework for Future De-Escalation Studies. J. Clin. Oncol..

[7] Ang K.K., Harris J., Wheeler R., Weber R., Rosenthal D.I., Nguyen-Tan P.F., Westra W.H., Chung C.H., Jordan R.C., Lu C. (2010). Human papillomavirus and survival of patients with oropharyngeal cancer. N. Engl. J. Med..

[8] Baumann M., Krause M., Overgaard J., Debus J., Bentzen S.M., Daartz J., Richter C., Zips D., Bortfeld T. (2016). Radiation oncology in the era of precision medicine. Nat. Rev. Cancer.

[9] Grégoire V., Langendijk J.A., Nuyts S. (2015). Advances in Radiotherapy for Head and Neck Cancer. J. Clin. Oncol..

[10] Nuyts S., Bollen H., Ng S.P., Corry J., Eisbruch A., Mendenhall W.M., Smee R., Strojan P., Ng W.T., Ferlito A. (2022). Proton Therapy for Squamous Cell Carcinoma of the Head and Neck: Early Clinical Experience and Current Challenges. Cancers.

[11] Vanderwaeren L., Dok R., Verstrepen K., Nuyts S. (2021). Clinical Progress in Proton Radiotherapy: Biological Unknowns. Cancers.

[12] Group. PTC-O. https://www.ptcog.ch/.

[13] Chaudhary P., Marshall T.I., Perozziello F.M., Manti L., Currell F.J., Hanton F., McMahon S.J., Kavanagh J.N., Cirrone G.A., Romano F. (2014). Relative biological effectiveness variation along monoenergetic and modulated Bragg peaks of a 62-MeV therapeutic proton beam: A preclinical assessment. Int. J. Radiat. Oncol. Biol. Phys..

[14] Matsumoto Y., Matsuura T., Wada M., Egashira Y., Nishio T., Furusawa Y. (2014). Enhanced radiobiological effects at the distal end of a clinical proton beam: In vitro study. J. Radiat. Res..

[15] Ilicic K., Combs S.E., Schmid T.E. (2018). New insights in the relative radiobiological effectiveness of proton irradiation. Radiat. Oncol..

[16] Lühr A., von Neubeck C., Krause M., Troost E.G.C. (2018). Relative biological effectiveness in proton beam therapy—Current knowledge and future challenges. Clin. Transl. Radiat. Oncol..

[17] Paganetti H. (2018). Proton Relative Biological Effectiveness—Uncertainties and Opportunities. Int. J. Part. Ther..

[18] Dok R., Nuyts S. (2016). HPV Positive Head and Neck Cancers: Molecular Pathogenesis and Evolving Treatment Strategies. Cancers.

[19] Gottgens E.L., Ostheimer C., Span P.N., Bussink J., Hammond E.M. (2018). HPV, hypoxia and radiation response in head and neck cancer. Br. J. Radiol..

[20] Wegge M., Dok R., Dubois L.J., Nuyts S. (2023). Use of 3D Spheroid Models for the Assessment of RT Response in Head and Neck Cancer. Int. J. Mol. Sci..

[21] Wang L., Han S., Zhu J., Wang X., Li Y., Wang Z., Lin E., Wang X., Molkentine D.P., Blanchard P. (2019). Proton versus photon radiation-induced cell death in head and neck cancer cells. Head. Neck.

[22] Sia J., Szmyd R., Hau E., Gee H.E. (2020). Molecular Mechanisms of Radiation-Induced Cancer Cell Death: A Primer. Front. Cell Dev. Biol..

[23] Huang R.-X., Zhou P.-K. (2020). DNA damage response signaling pathways and targets for radiotherapy sensitization in cancer. Signal Transduct. Target Ther..

[24] Wang L., Fossati P., Paganetti H., Ma L., Gillison M., Myers J.N., Hug E., Frank S.J. (2021). The Biological Basis for Enhanced Effects of Proton Radiation Therapy Relative to Photon Radiation Therapy for Head and Neck Squamous Cell Carcinoma. Int. J. Part. Ther..

[25] Wang L., Wang X., Li Y., Han S., Zhu J., Wang X., Molkentine D.P., Blanchard P., Yang Y., Zhang R. (2017). Human papillomavirus status and the relative biological effectiveness of proton radiotherapy in head and neck cancer cells. Head. Neck.

[26] Wang L., Yang L., Han S., Zhu J., Li Y., Wang Z., Fan Y.-H., Lin E., Zhang R., Sahoo N. (2020). Patterns of protein expression in human head and neck cancer cell lines differ after proton vs photon radiotherapy. Head. Neck.

[27] Chitsike L., Bertucci A., Vazquez M., Lee S., Unternaehrer J.J., Duerksen-Hughes P.J. (2023). GA-OH enhances the cytotoxicity of photon and proton radiation in HPV(+) HNSCC cells. Front. Oncol..

[28] Deycmar S., Faccin E., Kazimova T., Knobel P.A., Telarovic I., Tschanz F., Waller V., Winkler R., Yong C., Zingariello D. (2020). The relative biological effectiveness of proton irradiation in dependence of DNA damage repair. Br. J. Radiol..

[29] Görte J., Beyreuther E., Danen E.H.J., Cordes N. (2020). Comparative Proton and Photon Irradiation Combined with Pharmacological Inhibitors in 3D Pancreatic Cancer Cultures. Cancers.

[30] Vanderwaeren L., Dok R., Voordeckers K., Vandemaele L., Verstrepen K.J., Nuyts S. (2022). An Integrated Approach Reveals DNA Damage and Proteotoxic Stress as Main Effects of Proton Radiation in S. cerevisiae. Int. J. Mol. Sci..

[31] Chang C.-L., Lin K.-C., Chen W.-M., Shia B.-C., Wu S.-Y. (2024). Comparing the oncologic outcomes of proton therapy and intensity-modulated radiation therapy for head and neck squamous cell carcinoma. Radiother. Oncol..

[32] Hansen C.R., Jensen K., Smulders B., Holm A.I.S., Samsøe E., Nielsen M.S., Sibolt P., Skyt P., Elstrøm U.V., Nielsen C.P. (2024). Evaluation of decentralised model-based selection of head and neck cancer patients for a proton treatment study. DAHANCA 35. Radiother. Oncol..

[33] Mumaw D.A., Hazy A.J., Vayntraub A., Quinn T.J., Salari K., Chang J.H., Kalman N., Katz S., Urbanic J., Press R.H. (2024). Low contralateral failure rate with unilateral proton beam radiotherapy for oropharyngeal squamous cell carcinoma: A multi-institutional prospective study from the proton collaborative group. Radiother. Oncol..

[34] Orlandi E., Fontana G., Licitra L., Tinelli C., Camarda A.M., Grau C., Frank S.J. (2024). Comprehensive insights on the underlying potential and advantage of proton therapy over intensity-modulated photon radiation therapy as highlighted in a wide real world data analysis. Radiother. Oncol..

[35] Walser M.A., Bachmann N., Kluckert J., Köthe A., Tully C., Leiser D., Lomax A.J., Bizzocchi N., Langendijk J.A., Weber D.C. (2023). Clinical outcome after pencil beam scanning proton therapy and dysphagia/xerostomia NTCP calculations of proton and photon radiotherapy delivered to patients with cancer of the major salivary glands. Br. J. Radiol..

[36] Youssef I., Mohamed N., Kallini D., Zakeri K., Lin H., Han D., Qi H., Nosov A., Riaz N., Chen L. (2024). An Analysis of Positron Emission Tomography Maximum Standard Uptake Value Among Patients with Head and Neck Cancer Receiving Photon and Proton Radiation. Int. J. Radiat. Oncol. Biol. Phys..

[37] Youssef I., Yoon J., Mohamed N., Zakeri K., Press R.H., Chen L., Gelblum D.Y., McBride S.M., Tsai C.J., Riaz N. (2022). Toxicity Profiles and Survival Outcomes Among Patients with Nonmetastatic Oropharyngeal Carcinoma Treated with Intensity-Modulated Proton Therapy vs Intensity-Modulated Radiation Therapy. JAMA Netw. Open.

[38] Taku N., Wang L., Garden A.S., Rosenthal D.I., Gunn G.B., Morrison W.H., Fuller C.D., Phan J., Reddy J.P., Moreno A.C. (2021). Proton Therapy for HPV-Associated Oropharyngeal Cancers of the Head and Neck: A De-Intensification Strategy. Curr. Treat. Options Oncol..

[39] Szymonowicz K., Krysztofiak A., Linden J.V., Kern A., Deycmar S., Oeck S., Squire A., Koska B., Hlouschek J., Vüllings M. (2020). Proton Irradiation Increases the Necessity for Homologous Recombination Repair Along with the Indispensability of Non-Homologous End Joining. Cells.

[40] Fontana A.O., Augsburger M.A., Grosse N., Guckenberger M., Lomax A.J., Sartori A.A., Pruschy M.N. (2015). Differential DNA repair pathway choice in cancer cells after proton- and photon-irradiation. Radiother. Oncol..

[41] Di Pietro C., Piro S., Tabbì G., Ragusa M., Di Pietro V., Zimmitti V., Cuda F., Anello M., Consoli U., Salinaro E.T. (2006). Cellular and molecular effects of protons: Apoptosis induction and potential implications for cancer therapy. Apoptosis.

[42] Finnberg N., Wambi C., Ware J.H., Kennedy A.R., El-Deiry W.S. (2008). Gamma-radiation (GR) triggers a unique gene expression profile associated with cell death compared to proton radiation (PR) in mice in vivo. Cancer Biol. Ther..

[43] Narang H., Bhat N., Gupta S.K., Santra S., Choudhary R.K., Kailash S., Krishna M. (2009). Differential activation of mitogen-activated protein kinases following high and low LET radiation in murine macrophage cell line. Mol. Cell Biochem..

[44] Lerch S., Berthold S., Ziemann F., Dreffke K., Subtil F.S.B., Senger Y., Jensen A., Engenhart-Cabillic R., Dikomey E., Wittig A. (2020). HPV-positive HNSCC cell lines show strongly enhanced radiosensitivity after photon but not after carbon ion irradiation. Radiother. Oncol..

[45] Meerz A., Deville S.S., Müller J., Cordes N. (2021). Comparative Therapeutic Exploitability of Acute Adaptation Mechanisms to Photon and Proton Irradiation in 3D Head and Neck Squamous Cell Carcinoma Cell Cultures. Cancers.

[46] Vitti E.T., Kacperek A., Parsons J.L. (2020). Targeting DNA Double-Strand Break Repair Enhances Radiosensitivity of HPV-Positive and HPV-Negative Head and Neck Squamous Cell Carcinoma to Photons and Protons. Cancers.

[47] Lupu-Plesu M., Claren A., Martial S., N′Diaye P.D., Lebrigand K., Pons N., Ambrosetti D., Peyrottes I., Feuillade J., Hérault J. (2017). Effects of proton versus photon irradiation on (lymph)angiogenic, inflammatory, proliferative and anti-tumor immune responses in head and neck squamous cell carcinoma. Oncogenesis.

[48] Rieckmann T., Tribius S., Grob T.J., Meyer F., Busch C.J., Petersen C., Dikomey E., Kriegs M. (2013). HNSCC cell lines positive for HPV and p16 possess higher cellular radiosensitivity due to an impaired DSB repair capacity. Radiother. Oncol..

[49] Nickson C.M., Moori P., Carter R.J., Rubbi C.P., Parsons J.L. (2017). Misregulation of DNA damage repair pathways in HPV-positive head and neck squamous cell carcinoma contributes to cellular radiosensitivity. Oncotarget.

[50] Wang H., Wang B., Wei J., Meng L., Zhang Q., Qu C., Xin Y., Jiang X. (2020). Molecular mechanisms underlying increased radiosensitivity in human papillomavirus-associated oropharyngeal squamous cell carcinoma. Int. J. Biol. Sci..

[51] Sorensen B.S., Busk M., Olthof N., Speel E.J., Horsman M.R., Alsner J., Overgaard J. (2013). Radiosensitivity and effect of hypoxia in HPV positive head and neck cancer cells. Radiother. Oncol..

