# Supplementary material for: Radiobiology of Proton Therapy in Human Papillomavirus-Negative and Human Papillomavirus-Positive Head and Neck Cancer Cells
Source: Cancers (Basel). 2024 May 22;16(11):1959. doi: 10.3390/cancers16111959 (PMC11171379; doi:10.3390/cancers16111959)
Supplement: Supplementary file 1 [file cancers-16-01959-s001.zip › cancers-3003256-supplementary.pdf]

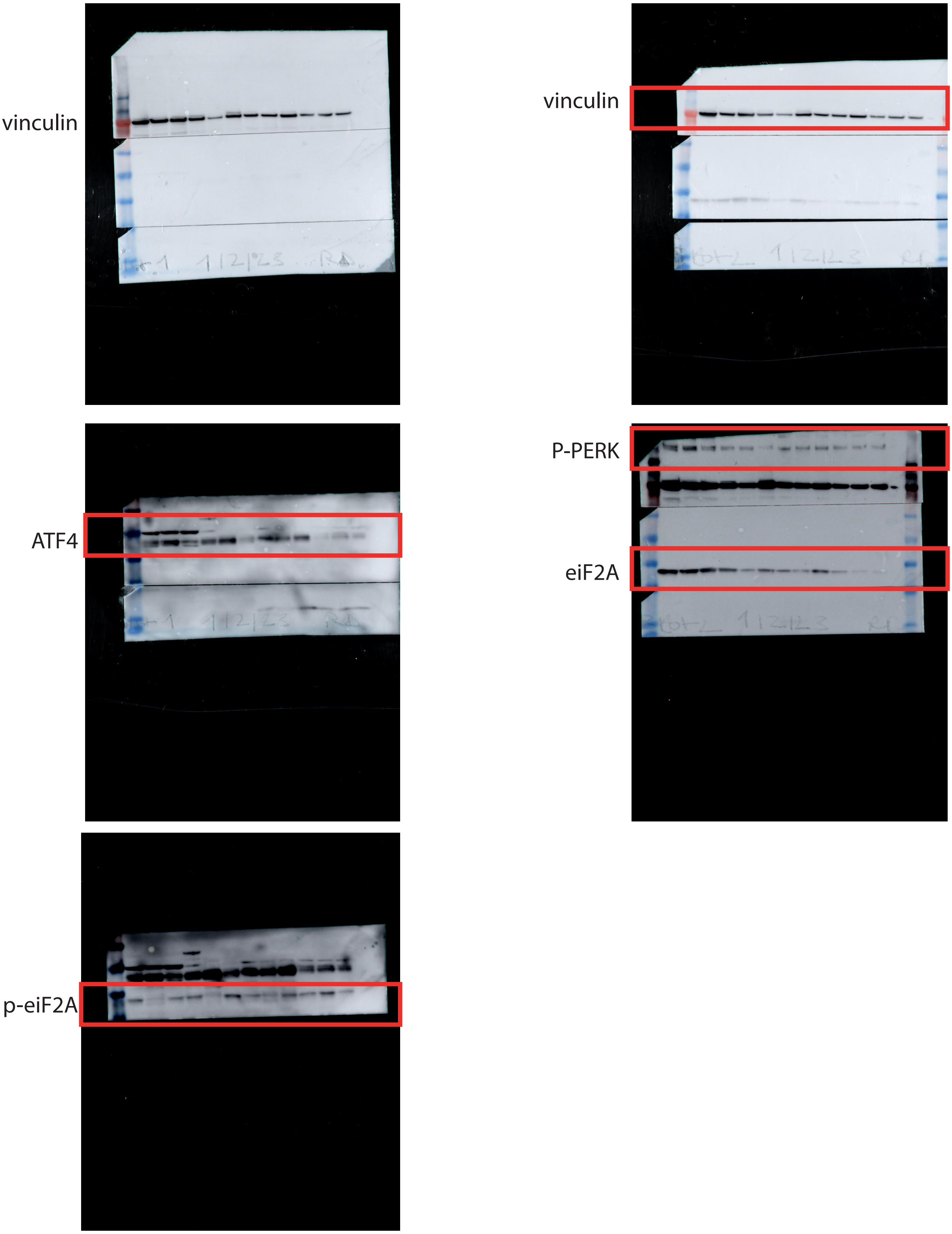

Figure S1: Uncropped western blot images of HNSCC cells treated with PT used in Figure 5.
Proteins used in Figure 5 are indicated with red boxes.
Protein ladder: SeeBlue™ (LC5925, Invitrogen, Carlsbad, CA, USA).

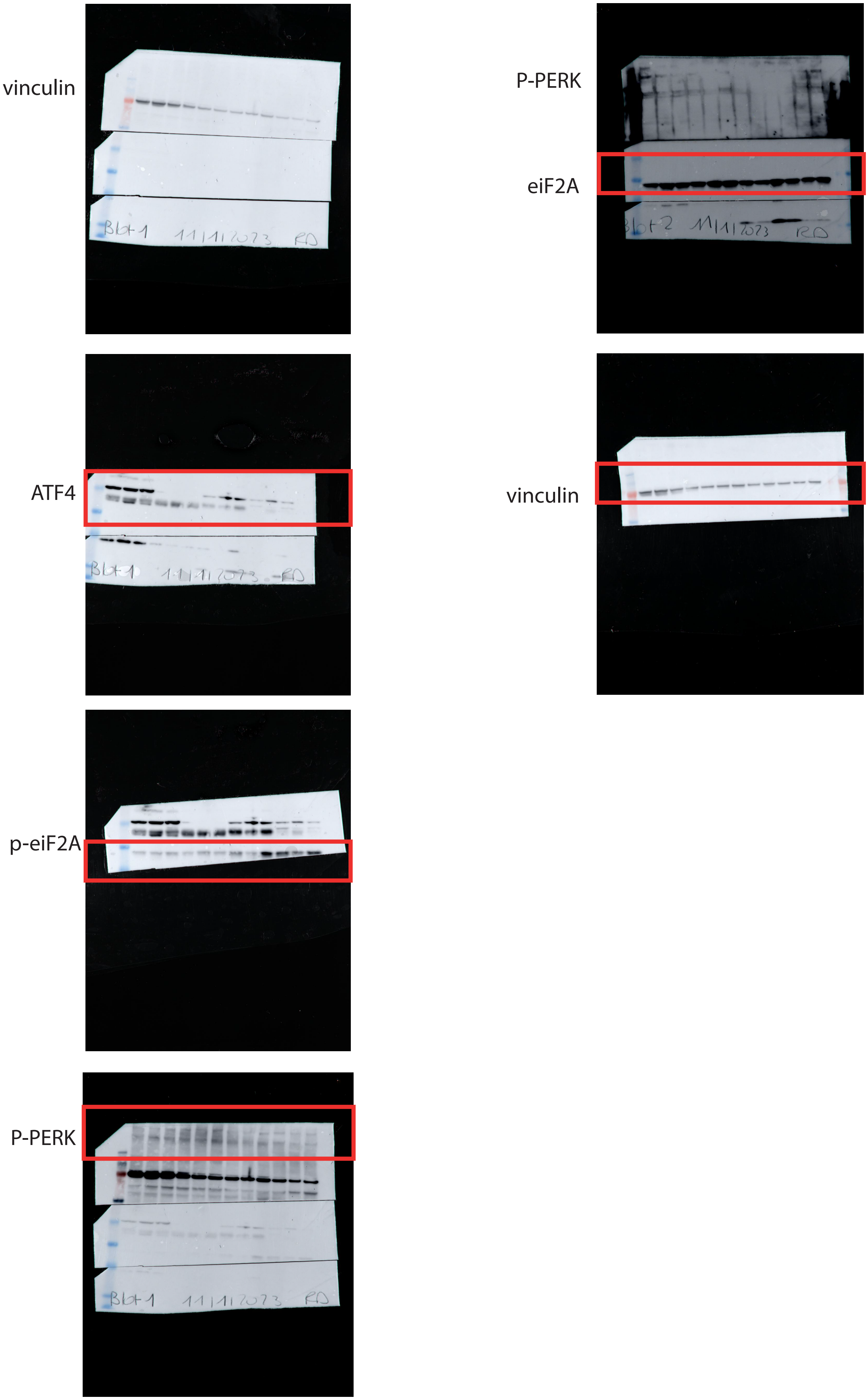

Figure S2: Uncropped western blot images of HNSCC cells treated with XRT used in Figure 5.
Proteins used in Figure 5 are indicated with red boxes.
Protein ladder: SeeBlue™ (LC5925, Invitrogen, Carlsbad, CA, USA).
